# Analysing a Chinese Regional Integrated Healthcare Organisation Reform Failure using a Complex Adaptive System Approach

**DOI:** 10.5334/ijic.2420

**Published:** 2017-06-19

**Authors:** Wenxi Tang, Lai Wei, Liang Zhang

**Affiliations:** 1 School of International Pharmaceutical Business, China Pharmaceutical University, CN; 2 School of Health Management, Zunyi Medical College, CN; 3 School of Medicine and Health Management, Tongji Medical College, Huazhong University of Science and Technology, CN

**Keywords:** Integrated Healthcare Organisation, Complex Adaptive System, health system reform, policy implementation, China

## Abstract

**Introduction:**

China’s organised health system has remained outdated for decades. Current health systems in many less market-oriented countries still adhere to traditional administrative-based directives and linear planning. Furthermore, they neglect the responsiveness and feedback of institutions and professionals, which often results in reform failure in integrated care. Complex adaptive system theory (CAS) provides a new perspective and methodology for analysing the health system and policy implementation.

**Methods:**

We observed the typical case of Qianjiang’s Integrated Health Organization Reform (IHO) for 2 years to analyse integrated care reforms using CAS theory. Via questionnaires and interviews, we observed 32 medical institutions and 344 professionals. We compared their cooperative behaviours from both organisational and inter-professional levels between 2013 and 2015, and further investigated potential reasons for why medical institutions and professionals did not form an effective IHO. We discovered how interested parties in the policy implementation process influenced reform outcome, and by theoretical induction, proposed a new semi-organised system and corresponding policy analysis flowchart that potentially suits the actual realisation of CAS.

**Results:**

The reform did not achieve its desired effect. The Qianjiang IHO was loosely integrated rather than closely integrated, and the cooperation levels between organisations and professionals were low. This disappointing result was due to low mutual trust among IHO members, with the main contributing factors being insufficient financial incentives and the lack of a common vision.

**Discussion and Conclusions:**

The traditional *organised health system* is old-fashioned. Rather than being completely organised or adaptive, the health system is currently more similar to a *semi-organised syste*m. Medical institutions and professionals operate in a middle ground between complete adherence to administrative orders from state-run health systems and completely adapting to the market. Thus, decision-making, implementation and analysis of health policies should also be updated according to this current standing. The simplest way to manage this *new system* is to abandon linear top-down orders and patiently wait for an explicit picture of IHO mechanisms to be revealed after complete and spontaneous negotiation between IHO allies is reached. In the meantime, bottom-up feedback from members should be paid attention to, and common benefits and fluid information flow should be prioritised in building a successful IHO.

## Introduction

Throughout the world, health systems face the ongoing predicament of being “isolated” and “fragmented”, which often results in inefficient resource utilisation and poor system performance [1]. To solve these problems, upon entering the 21^st^ century, integrated care organisation (IHO) reform has been recommended by the World Health Organization and many European countries [2,3]. Furthermore, collaboration among IHO members has been proven to be critical in forming a successful IHO in high-income countries [4,5,6,7,8]. However, in low- and middle-income countries (LMICs), especially those with less established market-oriented [9,10,11] national health systems, including China [12], desired goals are not achieved by many IHOs.

China launched its latest round of Healthcare System Reform in 2009, and has commenced Regional Integrated Healthcare Organisations (RIHO) in its urban and rural areas since 2012. An IHO is defined as an actual or virtual healthcare alliance with cooperative relationship among its members, which aims to provide continuous, coordinated and convenient service to residents [13]. In each pilot area, establishing at least one RIHO was recommended, with size varying according to population or number of medical institutions. Most RIHOs integrated community-based primary care with specialised services provided by “leading hospitals” in respective regions through technical agreements; some RIHOs also signed extended agreements to share financial incentives as well as the information system [14]. Most community facilities that provided primary care were still state-owned and professionals working there totally relied on national salaries for living; only a small number were funded by large hospitals after reform and partially shared medical savings in the RIHO.

This RIHO movement pinned the hope of “ice-breaking” by the central government’s series of reforms to reshape the healthcare delivery system, and has since then been given political priority by local governments as the preferred solution to improving quality of care and efficiency of health resources distribution. However, most RIHOs received no extra financial investment from the local government. On the contrary, the local government hoped that leading hospitals themselves would promote the integration for their own good. Current policy implementation in pilot regions has been proceeding clumsily, and the actual collaboration mechanism between different parts of the fragmented system remain untouched [15,16].

## Theory and Methods

We conducted a case study in Qianjiang, a rural district in southwestern China, whose IHO reform was initiated in May 2013. From 2013 to 2015, we observed the reform process and explored possible factors influencing policy implementation by investigating the behaviours of medical institutions and professional members in the IHO. We also evaluated the IHO type and found possible explanations from the perspective of system functioning for its current failure, and proposed a new semi-organised system and flow of policy implementation for the healthcare system reform.

## Setting

Qianjiang district is a typical rural area in China. Located in the south-eastern Chongqing municipality, it covers a mostly mountainous 2400 square kilometres; thus it has relatively low accessibility of healthcare compared with neighbouring areas. The district is served by two leading hospitals and 30 community health facilities, providing specialised and primary care to over 500,000 residents. Its poor public transportation brings much inconvenience for locals seeking proper treatment. Generally, it takes an average of 0.5 hours for rural patients to be seen by a doctor in primary care facilities, and then up to another 3 hours to travel from community-based facilities to the two hospitals located in the central district.

Doctors in community facilities provide simple treatment for common diseases, and only prescribe a limited number of drugs with economic efficiency named by the National Essential Drug List. To obtain a reliable diagnosis and more effective medication, rural patients have to travel to central hospitals. In 2008, the local government abolished compulsory regulations regarding gate-keeping and medical referral. Thus, patients did not have to first visit their community doctors to obtain approval before admission to central hospitals. This movement actually resulted in disorder of patient flow and chaos in the local medical market. Due to the weakening quality of rural care, a larger proportion of patients chose a large hospital as their first choice to receive care. However, this resulted in community facility medical costs blowing up to approximately 10 times or more compared with prior to 2008. This in turn placed high pressure both on the local medical insurance foundation and the patients themselves. Although the local health bureau had considered the long-term risks to the health system, their hands were tied when the free choices of people became deeply rooted.

## Qianjiang IHO

In May 2013, Qianjiang launched its IHO reform first in six pilot communities, and then promoted to cover the entire district. The reform carried multiple aims: first, to improve continuity of care by reconnecting communities and central hospitals, and meanwhile release more qualified health resources such as skilled personnel and techniques to remote areas; second, to decrease the proportion of large hospital visits and to then slow down growing medical expenditure; third, to improve quality of care through organisational cooperation and gradually restore confidence in community facilities among locals. After rounds of negotiations, the leading hospitals and 30 rural community facilities agreed on technical cooperation and shared responsibilities for all patients. Many of the agreements included the following: rebuild the referral system; offer extended services, technical assistance, training and further education; mutually approve diagnosis and treatment. The local government hoped that the contract would be signed spontaneously by the two sides without further financial investment, and that the IHO would offer a win-win solution for all regarding health system problems.

## Complex system and CAS theory

The IHO is typically a complex system [17]. Traditional organised and linear system theory has been outdated for at least 50 years in the healthcare area. Increasingly more academics have come to understand the complexity of this system and are thus looking for a better alternative. Within the IHO system, many medical institutions as well as individuals from different sections exist, and their adaptive behaviours have in turn made the system complex and unpredictable [18].

According to Mintzberg, who had founded his innovative theory of organisational strategies in 1983 and moved into the healthcare system area in the last decade, the modern health systems are usually composed of medical institutions that are simultaneously independent and interdependent, and the coordination and balance among these institutions are the very foundation for the successful functioning of the overall system [19]. Otherwise, uncoordinated behaviours of members will lower overall system performance, and poor system function will, in turn, impede maximising interest among members [20]. While division of labour is encouraged inside the system to achieve the benefit of specialization, an integrated approach is necessary to restore system connection and control over “isolated” members [21,22,23]. However, the individual connection was considered linear under the old-fashioned theory of an organised system; however it is nonlinear interaction among individuals which is responsible for the unforeseeable outcomes.

Fortunately, researchers have discovered complexity science, the “new science of the 21^st^ century” (as coined by Stephen William Hawking), which marks the new stage of system theory development [24]. The theory attributes the complexity of a system to the mass of information required for decision making and solution uncertainty brought about by “the amount and intensity of the internal communication among members” [25]. In a complex system, interactions between members are nonlinearly motivated and often bring about unpredictable chaos [26]. Therefore, in healthcare system reforms, complexity might lead to disturbance during policy implementation and to unexpected policy effects.

Among all emerging complex system theories, the Complex Adaptive System (CAS) is acknowledged to be the theory that has the most potential for studying the healthcare system and has also been extensively applied since the 21^st^ century [27,28,29]. The idea of CAS theory is as follows: (i) it is a “self-organised” system composed of several interacting subsystems, and each subsystem shares common interests through either competitive or cooperative ways; (ii) system members regarded as the adaptive agent can constantly optimise the system structure and their own behaviours through learning from each other and interacting with the environment; by their own initiatives, the adaptive members provide the basic motivation of evolution for the entire system; (iii) the system order is “endogenous”, so the whole system comprises sustainable adjusted organisms that adapt to environmental changes without the aid of external forces or the “highest role manager” [25,30,31,32,33]. By studying the CAS theory combined with the case observation, we proposed our approach for IHO systems.

## Case study

From May 2013 to May 2015, we observed the case and evaluated the effect of Qianjiang IHO reform. All data were collected through questionnaires and in-depth interviews with local health administrators and IHO members. The whole study proceeded through three stages.

First, we assessed the implementation of the Qianjiang IHO using a questionnaire to evaluate cooperative behaviour among organisational and individual members. Using D’Amour’s collaboration model, we examined nine types of organisational cooperative behaviours, to ensure collaboration mechanisms were built (see Table 1). We included questions to this questionnaire according to expert opinions, covering topics such as mutual trust, cultural cultivation, financial incentives, capacity development, clinical guidelines, team communications, and environment adaptability [34,35] (see Table 2). Then we investigated cooperation frequency and content inter-professionally using the questionnaire for individual members (see Table 3 and Table 4).

**Table 1:** Agreed items of collaboration before and after IHO reform.

	Formally Agreed	
	T = 0	T = 1

Referral System	20	24
Extended Specialty	10	12
Clinical Training	26	30
Technical Assistance	30	20
Mutual Recognition	30	21
Telemedicine	0	10 (6 suspended)
Further Education	19	25
Lectures	21	11
Academic Program	0	0

**Table 2:** Fulfilled factors of collaboration before and after IHO reform.

	Agency agreement	
	T = 0	T = 1

Culture	0	0
Leadership	0	2
Incentive Mechanism	0	2
Organized Team	0	4
Ability Training	20	15
Shared Techniques	6	4
Behavior Specification	0	4
Shared Information	0	3
Communication Tool	0	2
Stakeholder Negotiation	0	2

**Table 3:** Staff communication frequency (per month) before and after IHO reform.

	Community → County				County → Community			
	
	T = 0		T = 1		T = 0		T = 1	
	
	n	%	n	%	n	%	n	%

0	19	14.7	25	19.4	69	33.5	68	32.4
1	50	38.8	63	48.9	68	33.0	56	26.7
2	24	18.6	25	19.4	35	17.0	35	16.7
3	17	13.2	10	7.8	15	7.3	21	10.00
4 or above	19	14.7	6	4.7	19	9.2	30	14.3
	χ^2^ = 10.91, *P* = 0.0218				χ^2^ = 4.60, *P* = 0.331			

**Table 4:** Staff communication content before and after IHO reform.

	Community → County		County → Community	
	
	T = 0	T = 1	T = 0	T = 1

diagnosis	8	16	36	36
Exam result	38	48	72	69
Test result	39	50	73	72
Treatment	42	55	33	35
Medication	2	10	1	3
	χ^2^ = 21.08, *P* = 0.001		χ^2^ = 1.13, *P* = 0.890	

Second, we made a causal inference using the results from both the questionnaire and in-depth interviews (semi-constructed) to investigate the reasons for low cooperation performance and potential obstacles impeding IHO integration.

Finally, by induction, we proposed a *semi-organised system* explanation and developed a new theoretical framework to explain the flow of policy implementation using this approach. Then we confirmed a policy implementation procedure for healthcare reforms, and how to build a successful IHO in the healthcare system.

## Empirical Results

In all, 32 medical institutions and 344 professionals in the IHO were involved in the investigation. Generally, the Qianjiang IHO was “loosely integrated” rather than “closely integrated”, which was often the case in any other Chinese RIHOs. A close IHO was defined as integration which took substantial movements towards a merger or acquisition, or which was financially integrated; in a loose IHO the members were usually virtually bound by technical agreements concerning only technical support rather than financial assignment. The close IHO often proceeded with a combined payment reform that was approved by the local medical insurance authority [36]. Because there was no existing appraisal method or benchmark to evaluate an IHO, analysis covered aspects that are described below.

## Organisational collaboration

Directors of the investigated medical institutions completed the organisational questionnaire containing nine aspects that were recommended as being critical to organisational collaboration. T0 was the time when Qianjiang IHO was initiated, and the directors were asked to check off items that had already been conducted before IHO reform; T1 was May 2015, when the preliminary appraisal was done, and the directors checked off on what they thought had been actually realised according to the IHO contract (Table 1). Compared with the original contract, nearly no progress had been made after 2 years (χ^2^ = 5.842, *P* = 0.665).

Further inspection showed that, among 10 elements for a successful IHO that were recommended by the D’Amour model, progress was too little to be significant. Moreover, some factors even vanished after the reform (Table 2).

## Inter-professional cooperation

We examined professional communication as the most critical indicator for inter-professional cooperation. Clinical communication was defined as communication by professionals sharing information such as diagnosis and medication, which would help doctors from different member organisations to gain sound knowledge on patients’ previous treatments and reduce potential duplicate imaging examinations. Clinical communication not only reflected the intensity and frequency of the information exchanged among the IHO members, but more importantly, it reflected the possibility of providing continuous and coordinated treatment to patients during their referrals. Additionally, clinical communication contributed to the formation of group culture and mutual trust.

Among the 344 doctors who participated in the investigation, 129 were community staff and 215 were from county hospitals. We found that inter-professional cooperation was poor, and clinical communication and patient information sharing appeared unchanged after the reform. The majority of staff communicated less than three times a month with other members in the IHO. However, the case differed for members who initiated active communication. For example, community staff showed significant progress in terms of exchange frequency and content but their cooperative partners did not (Table 3 and Table 4).

## Causal inference for poor collaboration

To explore why organisational and inter-professional cooperation was poor under the current integration reform, we performed a causal investigation, and the results showed that the main obstacle was that mutual trust could not be formed within the IHO. Over 70% of the community and hospital staff showed conservative trust (*conservative trust* or below) in their partners. Among them, significantly more hospital doctors than community doctors held extreme distrust (*no trust* and *serious doubt*) (*p* < 0.05) (Table 5). This distrust would potentially devaluate the IHO, and result in unnecessary overuse of medical resources, which would ultimately aggravate patient burden.

**Table 5:** Mutual trust between community and hospital staff.

	Community → County		County → Community	

	n	%	n	%

No doubt	1	0.8	3	1.4
Trust	17	13.2	21	9.8
Conservative trust	88	68.2	124	57.7
No trust	19	14.7	37	17.2
Serious doubt	4	3.1	30	14.0

To further explore this trust problem, we asked doctors for their opinions regarding IHO failure. According to the D’Amour model, at least four aspects for successful collaboration are needed, and several elements that influence mutual trust for cooperation can be classified into these four categories: shared goals, financial interest and assignment, acknowledged common practice and group culture [37,38]. Moreover, trust risk from both sides was mainly due to lack of the following: financial incentives, sustainable cooperation mechanism, and effective communication platform (Table 6).

**Table 6:** Reasons for low mutual trust between community and hospital staff.

	Community staff		Hospital staff	

	n	%	n	%

Lack of sustainable cooperation mechanisms	202	58.7	101	78.3
Frozen practice position	144	41.9	40	31.0
Lack of financial incentives	233	67.7	97	75.2
Inconsistency of practice	106	30.8	36	27.9
Lack of continuous communication mechanisms	108	31.4	74	57.4
Lack of convenient communication tools	134	39.0	103	79.8
Lack of steady technical exchange	202	58.7	101	78.3
Other reasons	9	2.6	0	0

## Theoretical Analysis

We may infer from the empirical results that either the demands or the motivation of IHO members were sufficiently met. However, the responsiveness of system departments was ignored after policy orders were given. Because Qianjiang IHO is a typical case currently undergoing healthcare system reform, it gives us insight into how that kind of reform might often realise little desired effect. Traditional policy orders were given linearly in a top-down manner and were seldom adapted once assigned. In the organised system, feedback was often absent and information did not flow well. Therefore, when policy implementation is inherited via a traditional approach, the system could potentially collapse because of incompatible orders and ignorance of member reaction, thus resulting in failure of the IHO reform [39].

## A new health system perspective: “semi-organised system”

Traditionally, in the organised system, as long as principles are correct, a stable and predictable outcome should naturally ensue. When facing a changing environment, the top designer outside the system is responsible for taking action and developing countermeasures. Should the designer fail to respond to the environment, the system cannot change on its own and thus it collapses. Therefore, when unpredicted changes occur, the original guidelines are still imposed in a top-down manner, and the responsiveness and adaptability of the lower level of the system might strongly resist in a bottom-up direction. In this case, the whole system would be on the verge of breaking down because of top designers’ misjudgement. However, if no major defect is found within the decision-making process, the whole system becomes disoriented because the source of the problem cannot be identified. Any reform based on the old beliefs about the organisation might falter.

Furthermore, the failure of IHO reforms could be well explained by the complexity of the healthcare system. The medical institutions and professionals had their own purposes and initiatives and their behaviours constantly changed according to changes to their living conditions and instructions. By interacting with other members, they learned from each other and gained experience in how to better adapt to the environment, and hence sometimes violated the designed policy progress. Therefore, with or without the system reform and administrative orders, this adaptability of the members acted as the force driving the overall health system forward [40]. This explains why CAS theory is needed to creatively analyse the process of health policy implementation. In the modern healthcare system, time and space are no longer natural barriers to cooperation among members. What remains challenging is how to identify a positive interaction from the negative and then identify mechanisms to facilitate IHO cooperation [41].

**Figure 1:**
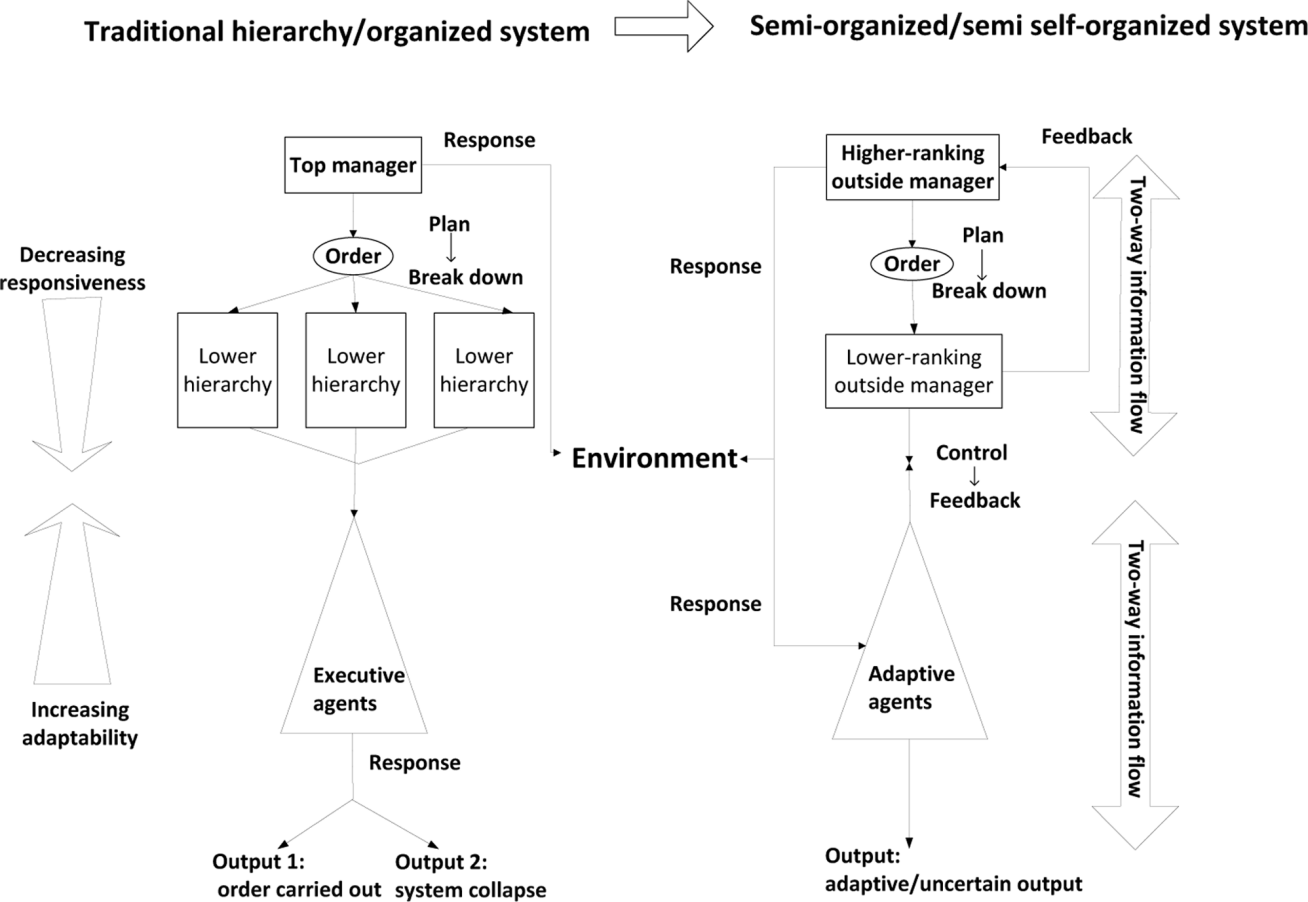
Traditional organised system and self-organised system approaches. Analysis flowchart of policy implementation following the semi-organised system approach.

From the analysis above, we could conclude that the traditional bureaucratic health system is an “*organised hierarchical system*” that ignores the adaptiveness of system members, and the complex adaptive system is “*self-organised*” and fully motivated by the initiatives of system members. Either theory suitably explains the healthcare system in less market-oriented countries. Therefore, we propose a “*semi-organised system*” perspective that combines both “organised system” and “adaptive system” approaches to substitute the old theory and to avoid over-interpreting member adaptiveness. For most countries with a national healthcare system, the system combines features from both organised and self-organised systems. Medical institutions are mostly self-organised and driven by their own interests, while decision-making and supervision is hierarchically organised and regulated [42]. Information exchanged in the system is in a dual direction, both top-down and bottom-up, and the decision-making process integrated with feedback from adaptive members in the healthcare system (Figure 1).

**Figure 2:**
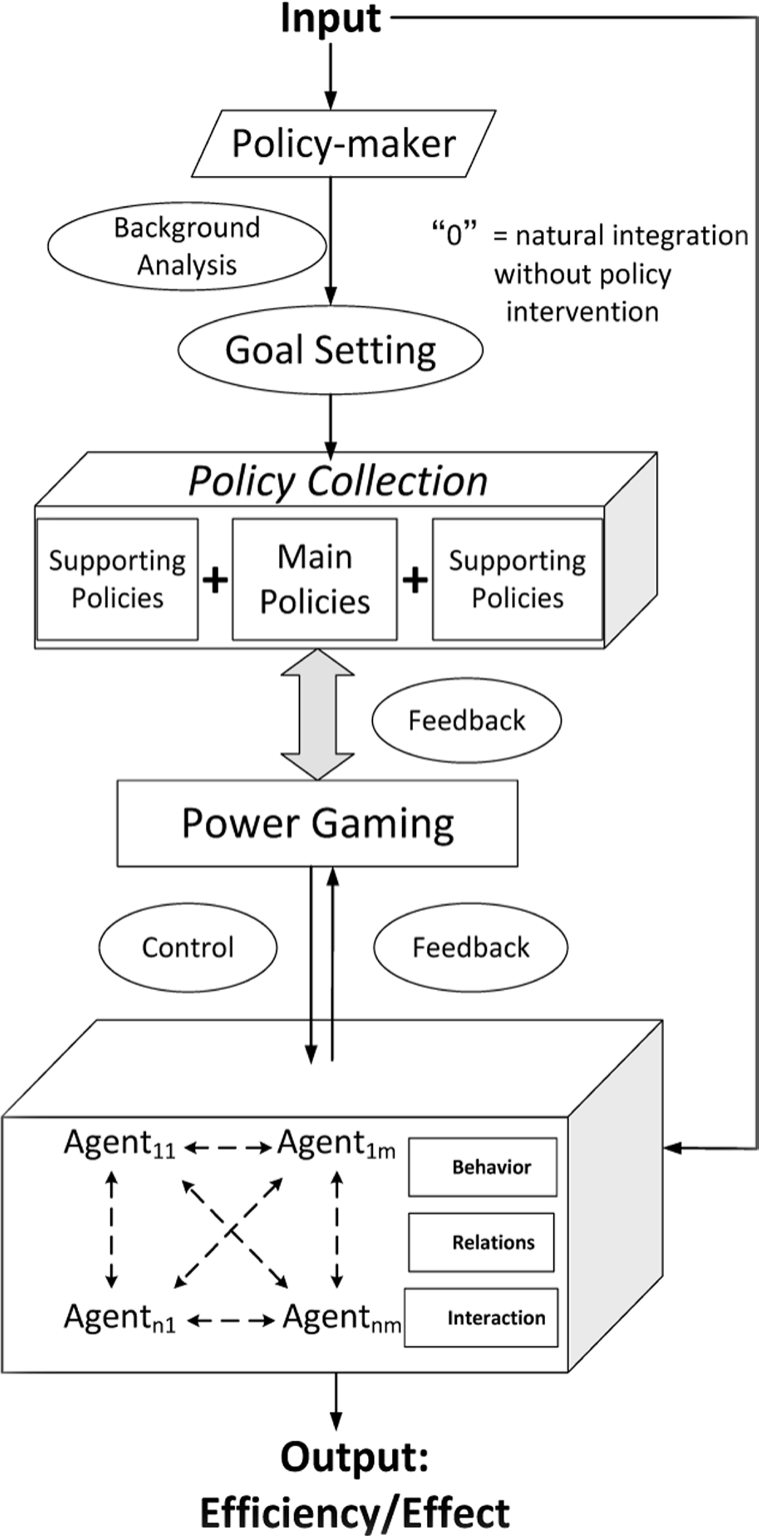
Model of policy implementation flow.

If a health system is considered semi-organised and contains many self-adaptive agents, then system members would act automatically by positively adapting their behaviours. Thus, the overall system would gain a certain ability of learning and interacting spontaneously in accordance with the environment, with continual adjustments made to the system structure as appropriate. Therefore, the system can respond to the environment much earlier than outside designers. The IHO reform, under the new approach, was an attempt to integrate the interests of various stakeholders, including the administrators, the hospital managers both from upper and lower levels, the professionals and the patients. Furthermore, policy implementation considered both administrative directives and spontaneous actions of IHO members. Thus, the paradigm for policy formulation and implementation process following the semi-organised system approach was proposed (Figure 2).

The policy implementation process included eight steps in this model: (1) Background analysis: analysing the environment, health demand, resource distribution, capacity and interaction of system members; (2) goal setting: setting desired policy goals and indicators for evaluation; (3) agent identification: identifying two types of agents, agents who execute policies and agents who control policy implementation; (4) decision of main policy: forming core items of the main policy that are directly implemented by execution agents after analysing and balancing responsibilities among system members; (5) decision to support policies: making arrangements for supporting policies such as payment and fiscal investment, which would potentially affect policy outcomes after considering the impact and costs involved for other administrative departments; (6) power gaming: games analysis among different system members, including policy-making departments, supporting departments, and execution and administrative agents; (7) feedback: receiving and absorbing feedback information from different members, then repeating steps 3 to 7 until an equilibrium appeared several rounds later; (8) control: comparing the predicted policy effect with the designed goals, revising the directives if possible and necessary and then inputting the revised directives into the system again.

## Stakeholder’s view of the semi-organised system perspective and implementation flow

We conducted an in-depth interview with 12 key stakeholders related to the IHO reform, collecting their opinions on the proposed approach and their preferred way of policy implementation (Table 7). For most interviewees, from either the administrative department or medical institutions, the healthcare system was a combination of “organised” and “self-organised” systems, and the preferred way of policy implementation was both “top-down” and “bottom-up”.

**Table 7:** Characteristics of key persons interviewed.

	Identity	Professional Years	Opinions	

			System-view	Implement-view

I	Health Administrator	21	Organized	Top-down
II	Health Administrator	15	Semi-organized	Mixed
III	Insurance Administrator	18	Self-organized	Mixed
IV	Insurance Administrator	8	Self-organized	Mixed
V	County hospital director	12	Semi-organized	Mixed
VI	County hospital professionals	14	Semi-organized	Bottom-up
VII	County hospital professionals	5	Self-organized	Mixed
VIII	Township hospital manager	10	Organized	Top-down
IX	Township hospital clinician	9	Self-organized	Bottom-up
X	Primary doctor	8	Semi-organized	Mixed
XI	Primary doctor	15	Organized	Mixed
XII	Village doctor	11	Self-organized	Mixed

## Discussion

### Suitability of the new system explanation

Mintzberg classified the organisations into three types: the mechanistic organisation, the professional organisation and the political organisation, according to “the coordination and control mechanism and the degree of power decentralization” [43]. In free market countries, the health systems are evolving toward becoming more flexible, however in less market-oriented countries like China and other nationally regulated health systems, the mechanistic organisation with typical bureaucratic departments is the most common case. However, from the perspective of CAS, the modern health systems are usually composed of multiple simultaneously independent and interdependent agents, and a balanced relationship among these agents is most critical for successful system functioning.

According to our perspective, the health systems are either totally organised or self-motivated. On the one hand, health resources are state-owned and will be distributed by the government every foreseeable period; on the other, the adaptiveness of system members is inherited, and no reform can succeed without acknowledging the free will of organisations and individuals. The only thing the government can do is be well aware of the complexity of the healthcare system, and learn to embrace the adaptability of system members. Additionally, the reform allows for mistakes, but we have to consider the affordability of error cost [44].

### Integration mechanisms in IHO

Key aspects to building a successful IHO are: First, fully recognise the relationship of system members, support the positive from the negative, and be ready to face conflict; second, try to avoid static thinking and work hard to achieve consensus among system members with wisdom; finally, give the different departments the right to freedom and to manage daily activities [45]. From successful experiences in some LMICs such as Korea [46], Singapore [47], India [48] and Ghana [49], we can well understand that building cooperative relationships by incorporating different organisations into an integrated delivery network is a beneficial mechanism. Furthermore, the cooperative relationship of IHO members should consider the following aspects: (1) recognition of gaming status of different agents; (2) shared vision and communication link between agents; (3) control and feedback; (4) mechanism of coordination between administrative and execution agents.

At least five elements should be provided to win mutual trust in an IHO: (1) shared motives, there must be sufficient financial incentive and agreed procedures for power gaming; (2) sustainable capacity cultivation, to cultivate staff capacities in developing positive cooperation and adapting to an evolving health system; (3) acceptable norms: implementing simple and definite clinical regulations and correcting potential deviations; (4) cooperation facilitators: convenient channels for information exchange to reduce the cost of formal communication among IHO members; (5) performance-based motivation: performance evaluation should be optimally quantified, strict and fair in accordance with original goals.

### Study limitations

The theoretical model proposed in this study can potentially be generalised to other less market-oriented countries. It can provide useful tools for analysing the process of health policy formulation, implementation and evaluation concerning IHO reforms involving multi-organisations.

However, the study also has some limitations: first, the evidence for IHO evaluation is weak. Because few direct measurements exist concerning how integrated an IHO can be, we chose D’Amour’s model of inter-professional collaboration to design the evaluation framework, however, further research on the IHO theory and evaluation tools for empirical studies is required. Second, no concrete definition or measurement exists for *adaptability* of system members in the healthcare area. Furthermore, evaluation of the policy implementation process relies on researcher experience and interviews with stakeholders, therefore the IHO appraisal in different countries cannot be conveniently generalised. Third, interaction among system members may be likened to a “black box”, and the actual adapting mechanism in social systems remains to be revealed. How to make the “black box” transparent remains unsolved, requiring further multi-disciplinary research [50].

## Conclusions

To summarise, the IHO reform in China has not achieved the desired effects of improving organisational cooperation; however, it has promoted cooperative motives of professionals from community facilities. Additionally, the IHO has failed to make a real connection between large hospitals and primary care facilities, and a low level of mutual trust is responsible for integration failure.

Theoretical analysis highlights that current health systems are challenged by many uncertainties that resemble “black boxes” in complex systems [51]. System members should be acknowledged as autonomous agents with independent purposes and adaptable ability. The traditional organised system and linear policy implementation should be modified with a semi-organised system and policy flow that might be more suited to realities that system members face. To date, IHO research needs further work regarding both study design and real world evidence, and testimonials from more cases in LIMCs using the semi-organised health system approach should be gathered for continual improvement.
